# Conventional techniques and emerging nanotechnologies for early detection of cancer metastasis via epithelial-mesenchymal transition monitoring

**DOI:** 10.1093/nsr/nwae452

**Published:** 2024-12-12

**Authors:** Zhen Zhang, Jing Wang, Alain Wuethrich, Matt Trau

**Affiliations:** Centre for Personalized Nanomedicine, Australian Institute for Bioengineering and Nanotechnology (AIBN), The University of Queensland, Brisbane, QLD 4072, Australia; Key Laboratory of OptoElectronic Science and Technology for Medicine, Ministry of Education, Fujian Provincial Key Laboratory for Photonics Technology, College of Photonic and Electronic Engineering, Fujian Normal University, Fuzhou 350007, China; Centre for Personalized Nanomedicine, Australian Institute for Bioengineering and Nanotechnology (AIBN), The University of Queensland, Brisbane, QLD 4072, Australia; Centre for Personalized Nanomedicine, Australian Institute for Bioengineering and Nanotechnology (AIBN), The University of Queensland, Brisbane, QLD 4072, Australia; School of Chemistry and Molecular Biosciences, The University of Queensland, Brisbane, QLD 4072, Australia

**Keywords:** nanotechnologies, epithelial-mesenchymal transition, cancer metastasis, biosensor, liquid biopsy

## Abstract

The epithelial-mesenchymal transition (EMT) is a critical process for cancer to metastasize by promoting invasiveness and dissemination of cancer cells in the body. Understanding and tracking EMT could improve cancer therapy by intervening in metastasis. Current approaches for investigating and detecting the EMT process often utilize traditional molecular biology techniques like immunohistochemistry, mass spectrometry and sequencing. These approaches have provided valuable insights into understanding signaling pathways and identifying biomarkers. Liquid biopsy analysis using advanced nanotechnologies allows the longitudinal tracking of EMT in patients to become feasible. This review article offers a molecular overview of EMT, summarizes current EMT models used in cancer research, and reviews both traditional techniques and emerging nanotechnologies employed in recent EMT studies. Additionally, we discuss the limitations and prospects of applying nanotechnologies in EMT research. By evaluating this rapidly emerging field, we propose strategies to facilitate the clinical translation of nanotechnologies for early detection and monitoring of EMT.

## Teaser

This review discusses how traditional methods for biomarker discovery and emerging nanotechnologies, particularly in liquid biopsy, enable EMT monitoring, offering new opportunities for the early detection of cancer metastasis.

## INTRODUCTION

Cancer metastasis remains the leading cause of cancer-related deaths globally [1]. Early detection of metastatic lesions significantly increases the chances of successful therapeutic intervention. However, identifying and treating metastatic lesions in their early stages presents a technical challenge [1,2]. Consequently, there is an urgent need for improved diagnostic and screening tools to identify metastatic lesions at an early stage in order to improve outcomes for patients.

**Figure 1.**
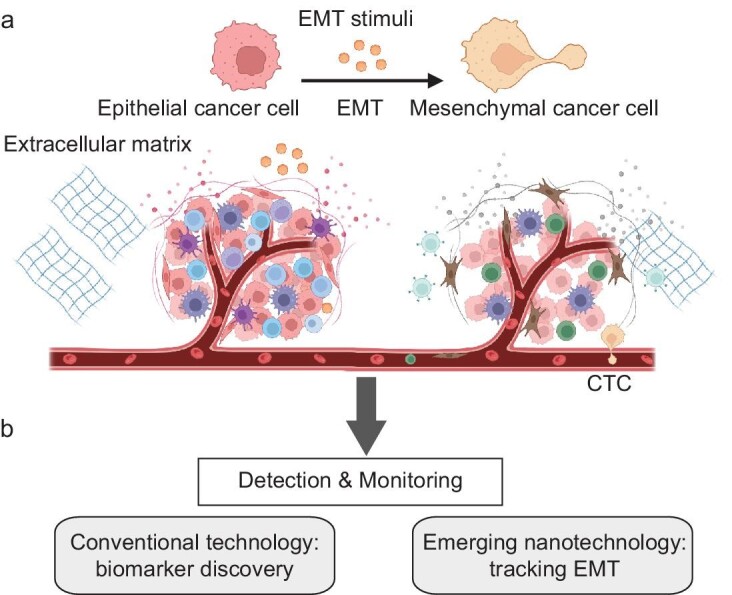
(a) The EMT process is stimulated by several stimuli, including growth factors, which cause epithelial cancer cells to lose connections to neighboring cells and extracellular matrix (ECM), leading to ECM degradation. This transition results in the formation of mesenchymal cancer cells (i.e. CTCs), facilitating their invasion into circulation and the establishment of secondary metastasis sites. (b) Conventional technologies and emerging nanotechnologies for EMT-related biomarker discovery and tracking the EMT process, respectively. Created in BioRender.

Epithelial-mesenchymal transition (EMT) is an essential process that initiates cancer progression and metastasis [3]. Most tumors originate from various types of epithelial cell types within the human body [4]. During tumor initiation and promotion, these cells can establish a local tumor microenvironment, comprising extracellular matrix (ECM) components, signaling molecules, and inflammatory conditions [5]. Changes in the tumor microenvironment can activate the EMT process (Fig. 1a), including increasing extracellular signals (such as growth factors), altering inflammatory conditions, and inducing pathologic states like hypoxia [6]. During the EMT process, epithelial cancer cells detach from neighboring cells and ECM, leading to ECM degradation and the acquisition of mesenchymal characteristics, such as increased motility and invasiveness. As these cells undergo EMT, they often enter the circulation and are referred to as circulating tumor cells (CTCs) (Fig. 1a) [4,7]. CTCs play a crucial role in metastatic dissemination by traveling through the bloodstream or lymphatic system to distant sites, where they can colonize and form secondary tumors [8]. The detection and characterization of CTCs have emerged as important biomarkers for monitoring cancer progression, assessing the risk of metastasis, and evaluating therapeutic efficacy. EMT also induces phenotypic plasticity in carcinoma cells, often associated with stemness, self-renewal, and therapy resistance, thus complicating cancer management [9–11]. Investigating and detecting EMT offer potential strategies for early detection, therapeutic intervention, monitoring, and prevention of metastasis, ultimately improving patient prognosis.

EMT is a dynamic process that is difficult to monitor longitudinally. Current methods for detecting EMT involve laboratory molecular analysis of EMT-related proteins and transcription factors using techniques such as western blot, real-time quantitative PCR (qPCR), and immunohistochemistry (IHC) [12–14]. However, these methods may struggle to capture temporal changes in the tumor microenvironment effectively. To address these limitations, advanced technologies have been developed to track the EMT process more accurately and in real time.

Liquid biopsy, which analyzes tumor-related biomarkers in bodily fluids, has emerged as an important source for cancer biomarkers and has shown significant potential in monitoring EMT. The advantages of liquid biopsy include its minimally invasive nature and its capability for routine sample collection, facilitating real-time monitoring of the EMT process [15,16]. However, detecting liquid biopsy markers such as CTCs, soluble proteins, exosomes or extracellular vesicles (EVs) and nucleic acids is challenging due to their rarity and heterogeneity [16]. The development of nanotechnologies has significantly enhanced liquid biopsy analyses [17]. Despite numerous excellent reviews on liquid biopsy biosensors, a comprehensive focus on their applications in EMT monitoring is lacking. To address this gap, we present an extensive review of the EMT mechanism and associated signaling pathways, current models for EMT study, and conventional and emerging liquid biopsy-based nanotechnologies for EMT tracking (Fig. 1b). We also identify the existing challenges impeding the translation of these emerging technologies into clinical applications.

## EMT

### EMT mechanisms

**Figure 2.**
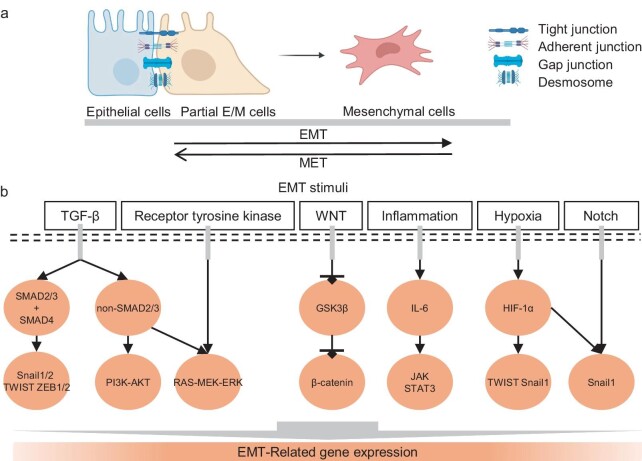
EMT process and stimuli. (a) EMT involves the morphological and phenotypical transformation of epithelial cancer cells into mesenchymal cancer cells. Conversely, MET refers to the reversal of mesenchymal cells back to epithelial cells. (b) Major EMT stimuli and corresponding signaling pathways include TGF-β, RTK, Notch, Wnt, inflammation, and hypoxia-induced signaling pathways.

EMT is one of the significant cellular phenotypic transformations [3,18–20], which is reversible and transient (Fig. 2a) [3,19,21,22]. During the epithelial stage, cells maintain apical-basal polarity through epithelial cell-cell and cell-ECM connections [18,23,24]. These cellular connections are formed by tight junctions, cadherin junctions (involving E-cadherin connections to the actin cytoskeleton), gap junctions, and desmosomes (Fig. 2a) [18,25]. Epithelial cells also anchor to the ECM through integrin, forming cell-ECM connections.

During the EMT process, epithelial cells lose connections with neighboring cells and the ECM by triggering biochemical changes, including: (1) the downregulation of epithelial markers (e.g. E-cadherin and epithelial cell adhesion molecule, EpCAM); (2) the downregulation of tight junction proteins; and (3) the upregulation of mesenchymal markers (e.g. vimentin, N-cadherin and fibronectin). These biochemical changes ultimately cause the cells to adopt a spindle-shaped morphology (Fig. 2a) [18,19].

After transitioning to mesenchymal cells, which are typically individualized and exhibit distinct interactions with the ECM, these cells can enhance the production of matrix-degrading metalloproteinases (MMPs) and remodel the ECM to facilitate cell movement [25,26]. Many studies suggest that EMT is linked to profound epigenetic changes associated with cell states and microenvironmental alterations, such as cytokines, hypoxia, and inflammation (Fig. 2b) [18,19,22,27,28].

### EMT stimuli and related signaling pathways

EMT is influenced by multiple signaling pathways and is typically initiated by EMT-related transcription factors (e.g. Snails, Slug, zinc finger E-box-binding homeobox 1 and 2 (ZEB1/2), and Twist), which suppress the expression of adherent and tight junction molecules [18,29,30]. These factors are activated through various EMT stimuli such as cytokines, inflammation, and hypoxia (Fig. 2b) [18,19,22,27,28].

The major stimuli for EMT include autocrine factors such as members of the transforming growth factor-β (TGF-β) family and epidermal growth factor (EGF) family [31]. In the TGF-β-induced EMT signaling pathway, TGF-β binding initiates the phosphorylation of TGF-β receptor I, leading to the recruitment of SMAD2/3 and formation of a complex with the coactivator SMAD4. This complex translocates into the nucleus, where it binds to regulatory elements and activates transcription factors (such as Snail1/2, Twist, and ZEB1/2) [26,32,33]. These transcription factors then bind to the promoter region of epithelial genes, such as *CDH1*, leading to the inhibition of E-cadherin expression and disruption of adherent junctions [18,26]. Additionally, TGF-β induces non-SMAD pathways, including PI3K–AKT and Ras-MEK-ERK, both of which contribute to EMT. Similarly, EGF induces the EMT process through the receptor tyrosine kinase (RTK) pathway, followed by activation of the Ras-MEK-ERK pathway and expression of EMT-related proteins [18,26].

The Wnt signaling pathway is another major contributor to EMT. When Wnt binds to its receptor, it inhibits the enzyme glycogen synthase kinase 3 β (GSK3β), preventing the degradation of β-catenin. This allows β-catenin to accumulate and translocate to the nucleus, where it induces the expression of EMT-related genes [26,34–36]. Additionally, the inhibition of GSK3β increases the stability of Snail, further promoting the EMT process [31,37].

Microenvironment changes can act as stimuli for EMT. For instance, inflammatory signals such as interleukin-6 (IL-6) can regulate Snail expression through the JAK–STAT3 pathway [38]. In hypoxic conditions, hypoxia-inducible factor 1α (HIF-1α) binds directly to the Twist promoter, promoting EMT [39]. Additionally, HIF-1α interacts with Notch signaling, leading to the up-regulation of Snail1 expression [22,40–42]. These signaling pathways collectively shape the EMT process by regulating the expression of EMT-related genes.

## EMT MODELS IN CANCER RESEARCH

To comprehensively investigate the mechanism underlying EMT in cancer metastasis and develop potential therapeutic strategies, establishing an effective EMT model in cancer research is essential. This section introduces various models including cell line models, animal models, and tumor-on-a-chip models, designed for studying the EMT process. Utilizing these models can provide a more detailed understanding of the EMT mechanism and aid in developing targeted interventions against cancer metastasis.

### Cell line models

One effective approach to studying the EMT process in cancer metastasis is using tumor cell line models. The first tumor cell line model for EMT study was established using TGF-β as the inducer [43–45]. Other cytokines such as IL-6 [38], IL-1β [46], and IL-17α [47] have also been used to stimulate EMT in tumor cell line models. Additionally, the hypoxia-induced EMT model has been well-established in multiple tumor cell lines [48,49]. These cell line models prove to be valuable for investigating changes in EMT-related marker expression (e.g. E-cadherin, Vimentin, *etc*.) during cancer development and for discovering new signaling pathways and transcription factors associated with EMT.

An illustrative example of investigating EMT-related marker expression in tumor cell line models dates back to the early 1990s. During this period, researchers examined the downregulation of E-cadherin expression, which is associated with increased invasion in breast, lung, and pancreatic epithelial tumor cell lines [50,51]. Notably, these studies observed a reversal of cell invasion phenotype after transfection with E-cadherin cDNA, marking the pioneering discovery that E-cadherin acts as a suppressor of invasion in cancer. In recent years, the American Type Culture Collection (ATCC) has developed an advanced *in vitro* model for basic research and the discovery of new anti-EMT drugs. For example, CRISPR/Cas9 gene editing was used to generate a vimentin red fluorescent protein (RFP) fusion reporter in a non-small cell lung cancer (NSCLC) cell line for real-time tracking of EMT status, resulting in the discovery of two EMT inhibitors [52]. Additional *in vitro* models, combined with recent technologies, will further be discussed in next section.

### Animal models

Animal models play a pivotal role in advancing our understanding of the molecular mechanisms involved in EMT [53,54]. These models enabled the investigation of EMT in cancer metastasis and supported the development of *in vitro* studies.

One notable model is using transgenic mice, such as those involving the deletion of *CDH1* (i.e. *E-cadherin* gene). Perl *et al.* using a Rip1Tag2 transgenic mouse model with the deletion of *CDH1*, provided the first *in vivo* evidence of EMT's involvement in cancer metastasis [55]. They observed that the loss of E-cadherin led to the enhanced invasiveness of cancer cells and caused cancer progression. Another study utilized a Cre-lox-based mouse model of pancreatic ductal adenocarcinoma (PDAC) and identified that invading cells exhibit EMT. Importantly, this study also highlighted the role of inflammation in driving the EMT process, suggesting that targeting inflammatory pathways could potentially inhibit EMT and reduce metastatic spread [53].

Further support for the connection between EMT and cancer metastasis comes from studies by Ye *et al.* [54]. By injecting cancer cells with fluorescently labeled Snail1 into the tail vein of MMTV-PyVT transgenic mice, real-time tracking of EMT *in vivo* was achieved. This model was used to identify the role of Snail1-postive cancer cells in promoting metastasis to the lungs and establish the link between EMT markers and metastatic potential.

In another innovative approach, Jimenez *et al.* [56] established an EMT model using transgenic zebrafish. This approach involved inserting the Snail1 promoter into a green fluorescent protein (GFP) plasmid to generate a transgenic strain serving as an EMT reporter. The reporter was transfected into neural crest cells before delamination from the neuroectoderm, enabling observation of the EMT process and migration using a fluorescent microscope.

The reversibility of the EMT process has been investigated using mouse models. Tsai *et al.* [57] observed Twist1 expression during the EMT process and noted that the subsequent reversion of EMT occurred at a distant site upon deactivation of Twist1 expression. This reversibility demonstrates the plasticity of EMT and provides therapeutic potential to limit cancer metastasis.

In summary, animal models provide a direct and effective means to study the EMT process, offering valuable insights, particularly for drug screening of EMT-related pathways inhibitors.

### Tumor-on-a-chip model

**Figure 3.**
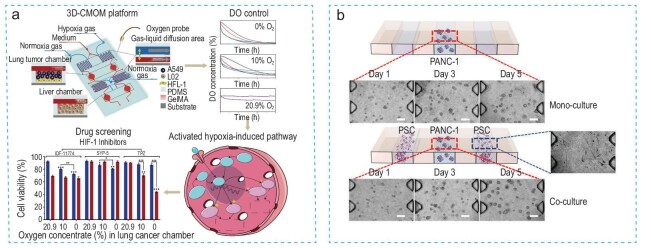
Tumor-on-a-chip for investigating the EMT process. (a) A three-dimensional-culture multiorgan microfluidic (3D-CMOM) platform used to detect the EMT process and to study hypoxia-induced lung cancer metastasis. Reproduced with permission from Ref. [59]. Copyright 2021, American Chemical Society. (b) Pancreatic stellate cells cultured in a 7-channel microchannel plate within a 3D-collagen matrix to mimic the *in vivo* tumor microenvironment and recapitulate the EMT process. Reproduced with permission from Ref. [61]. Copyright 2018, Springer Nature.

The tumor-on-a-chip platform represents an advanced *in vitro* study method that has gained significant attention in recent years. These platforms are used for three-dimensional (3D) tissue culture, effectively mimicking organ structures and recapitulating crucial biological parameters and functions observed *in vivo*. They hold the potential to predict clinical trial outcomes more accurately by streamlining cancer drug screening. Additionally, these platforms can replicate patient-specific, organ-level cancer pathophysiology and responses to therapy, enabling the investigation of EMT mechanisms and the development of new therapeutic strategies to intervene in metastasis [58]. In the work by Zheng *et al.* [59], a 3D-culture multi-organ microfluidic (3D-CMOM) platform was established to mimic the EMT process for studying hypoxia-induced lung cancer metastasis. By precisely controlling the dissolved oxygen concentration in cell chambers (Fig. 3a), the researchers created a hypoxic microenvironment. This environment led to an elevated level of HIF-1α in cancer cells, which correlated with increased expressions of Snail and Slug, resulting in cancer metastasis. Subsequently, they applied HIF-1α inhibitors to the cancer cells and observed enhanced treatment effects using the platform.

Arai *et al.* [60] developed a 3D screening system with specialized NanoCulture plates containing nano-scale grid scaffolds. This design reduced cell adhesion, promoted migration, and facilitated 3D spheroid formation. They subsequently used this system as a drug screening tool, screening 1330 drug candidates and identifying at least two compounds with potential EMT inhibitory activity. Traditional biological analyses, such as qPCR, western blot, and live imaging techniques were then used to characterize the performance of these EMT inhibitors.

The co-culture of 3D tumor spheroids with fibroblasts has emerged as another valuable *in vitro* model for EMT study [61,62]. Lee *et al.* [61] cultured pancreatic stellate cells in a 7-channel microchannel plate within a 3D-collagen matrix to mimic the *in vivo* tumor microenvironment and recapitulate the EMT process (Fig. 3b). The increased EMT-related markers identified in this setup indicated the stimulation of the EMT process, emphasizing the potential of the microfluidic co-culture method for studying EMT and drug resistance development. Meanwhile, Kim *et al.* [62] cultured cancer cells with cancer-associated fibroblasts on collagen gel, observing cross-talk between these cells that result in increased mesenchymal markers and reduced expression of epithelial markers.

However, there are technological limitations that need to be addressed before tumor-on-a-chip models can transition to a clinical research setting. These include isolating all relevant cell types (cancer cells, endothelial cells, stromal cells, and immune cells) from the same patients and constructing organ chips with the appropriate cell types, proportions, and locations to accurately mimic *in vivo* behaviors and responses. Additionally, establishing cross-talk between these cell types presents challenges, requiring significant time and careful optimization [58].

## CONVENTIONAL MOLECULAR TECHNIQUES FOR DETECTING AND MONITORING EMT

In the traditional biological field, significant attention has been directed toward exploring the signaling trajectories and markers associated with the EMT process. The investigation of molecular biology often employs widely recognized techniques such as western blot, immunohistochemistry, mass spectrometry, sequencing methods, and fluorescence labeling. These conventional methods have long been the cornerstone of biological research, allowing researchers to study the molecular foundation governing the EMT process. Additionally, spatial transcriptomics is a recently developed approach that enables the detection of transcription levels within tissue. Some studies have utilized this novel method to trace the EMT process in tumor tissue. In the subsequent sections, we will review how traditional biological methods have been applied to monitor the EMT-associated biological process.

### Sequencing and bioinformatic analysis

**Figure 4.**
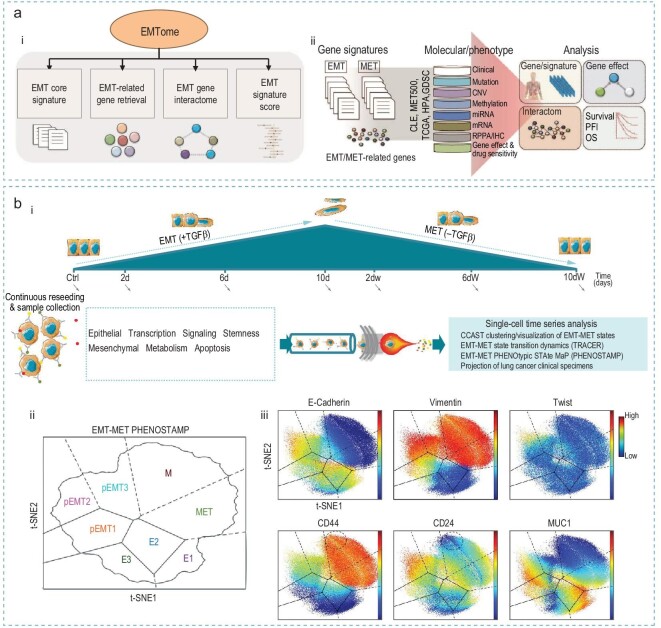
Transcriptomic and proteomic analyses of EMT. (a) Overview of the EMTome for sequencing: (i) schematic of the EMTome database, and (ii) workflow to generate the EMT-signature score. Reproduced with permission from Ref. [63]. Copyright 2020, Springer Nature. (b) Mapping the EMT process based on mass cytometry data: (i) workflow of cells receiving TGF-β treatment time-course; (ii) a reference map of EMT-MET states; (iii) expression profiles of six clustering markers in lung cancer cell lines (HCC827) visualized on the EMT-MET reference map. Reproduced with permission from Ref. [65]. Copyright 2019, Springer Nature.

Sequencing technology has become one of the most crucial analytical tools for studying EMT in cancer at the molecular level. Its application extends predominantly to investigating the mechanism and signaling pathways underlying the EMT process. Vasaiker *et al.* [63] developed the EMTome, a comprehensive database that integrates: (i) EMT gene signatures; (ii) multiomics features of EMT-related genes across diverse cancer types; (iii) interactomes involving EMT-related genes; (iv) immune profiles identified from The Cancer Genome Atlas (TCGA) cohorts through transcriptomics, epigenomics, and proteomics; and (v) correlations with drug sensitivity and clinical outcomes in cancer cohorts associated with EMT gene signatures (Fig. 4a(i)). By selecting 314 genes as the EMT/MET core signatures from a pool of 4499 publications and collecting RNA expression datasets from the Cancer Cell Line Encyclopedia as well as patient genomic, epigenetic, and transcriptomic data from 32 TCGA cancer types, they generated an EMT-signature score. This score has proven valuable in quantifying the level of EMT progression and exploring related signaling pathways (Fig. 4a(ii)).

Song *et al.* [64] conducted transcriptome sequencing (RNA-seq and microRNA-seq) in an *in vitro* lung cancer model to simulate the EMT process in patients. They identified three novel and early EMT markers (ALNT6, SPARC, and HES7) that can be upregulated to respond to cancer microenvironmental stimuli within 3 h, triggering further EMT processes. These markers were subsequently validated in lung cancer patients and may serve as targets for future drug screening efforts.

While sequencing methods are the predominant approach for discovering new markers and signaling pathways in the EMT process, these methods are limited in their ability to monitor the dynamic shifts of EMT in real time. Additionally, most of these discoveries remain confined to the laboratory setting due to the extensive database analysis and sample size requirements needed to achieve ultrasensitive levels. Consequently, translating these findings into clinical applications requires further evaluation, particularly in developing targeted therapies aimed at inhibiting the EMT process and intervening in cancer metastasis.

### Mass spectrometry

Mass spectrometry can be applied to study EMT in cancer by precisely identifying and quantifying proteins and metabolites involved in the process. This technique allows for detailed analysis of cellular changes, offering insights into the molecular mechanisms driving EMT and potential therapeutic targets. Karacosta *et al.* [65] employed mass cytometry time-course analysis and the computational tool to construct EMT to MET trajectories in NSCLC. They monitored the evolution in the proteomic expression levels of EMT-related markers during TGF-β-treatment (Fig. 4b). Based on the levels of E-cadherin, Vimentin, CD44, CD24, Twist, and MUC1, they defined eight distinct EMT and MET states: E1, E2, E3, pEMT1, pEMT2, pEMT3, M, and MET. The study demonstrated EMT is plastic among states and not a single trajectory involved in the spectrum. The algorithm developed in this study identified multiple trajectories, significantly distinguishing EMT and MET states and revealing bidirectionality between certain states. The map showing the expression levels of these six markers across different EMT states are shown in Fig. 4b(ii, iii).

They further successfully applied their approach to determine the spectra of EMT and MET states in five NSCLC tissue samples. While the study provided a framework to facilitate phenotypic analysis of clinical samples related to the EMT and MET trajectory, offering insights into the clinical relevance of EMT in cancer, one limitation was the relatively small clinical sample size. Further studies with a larger sample size are essential to solidify the conclusions. Nevertheless, their methods have significantly enhanced the understanding of the EMT–MET spectrum at the proteomic level and have proven valuable in interpreting corresponding mutations in clinical specimen data.

### Gene fusion

In addition to analyzing the EMT transcriptomic and proteomic trajectories during the EMT process, gene fusion technology has been applied to enable dynamic visualization of the EMT process in living cells. Vimentin, an EMT-related marker, is overexpressed during the EMT process. Targeting vimentin at both mRNA and protein levels could help to detect the EMT process and aid in diagnosing metastatic stages of epithelial cancers. However, imaging this intracellular protein in living cells poses a challenge.

**Figure 5.**
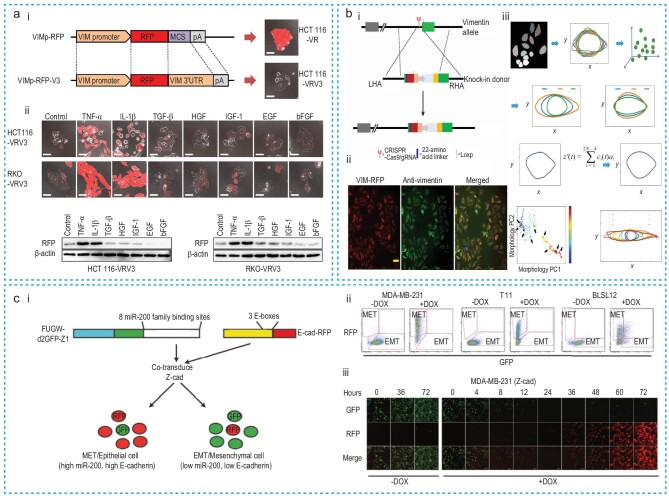
Immunostaining and imaging techniques. (a) (i) Structure for VIM promoter-driven RFP expression vector. (ii) Fluorescence images of HCT116 and RKO cells with different EMT stimuli for 48 h. Reproduced with permission from Ref. [66]. Copyright 2019, Springer Nature. (b) (i) The generation of VIM-RFP knock-in allele. LHA: left homology arm; RHA: right homology arm. (ii) Colocalization immunostaining of VIM and endogenous VIM-RFP (scale bar 20 μm). (iii) Quantification of single-cell trajectories based on cell morphology. Reproduced with permission from Ref. [67]. Copyright 2020, The American Association for the Advancement of Science. (c) (i) The Z-cad dual sensor is transduced to cells, which detects cellular changes of miR200, and E-cadherin based on their expression of GFP and RFP proteins. (ii) Flow cytometry results of MDA-MB-231, T11, and BLSL12 MET and EMT populations after treating with doxycycline (DOX). (iii) Confocal fluorescence microscopy of MDA-MB-231 cells imaging after DOX induction. Reproduced with permission from Ref. [68]. Copyright 2016, Springer Nature.

Maier *et al.* [65] developed an innovative intrabody (chromobody)-based imaging approach to study the spatiotemporal organization of endogenous vimentin upon EMT induction. They created the vimentin chromobody by fusing a vimentin-specific nanobody with green fluorescence, allowing for the visualization of endogenous vimentin in living cells. This approach revealed that the apical-basal polarity of epithelial cells shifted to the front-rear polarity of mesenchymal cells, as observed under a confocal microscope. Takeshi Ieda *et al.* [66] developed a fluorescence imaging-based EMT monitoring system by embedding the RFP into the mesenchymal marker vimentin (VIM) gene, with or without 3′-untranslated region (3′-UTR) (Fig. 5a(i)). They found that VIM 3′-UTR-carrying cells (HCT116VRV3) were able to undergo the EMT process in response to inflammatory microenvironment stimuli (Fig. 5a(ii)). This method further supports the dynamic study of EMT in living cells, enhancing our understanding of the process and its implications in cancer metastasis.

Similarly, Wang *et al.* [67] developed an endogenous fluorescent labeling-based imaging approach to track EMT-related changes in cellular status (Fig. 5b). This technique minimizes perturbation to cell physiology and provides live-cell images of high-dimensional morphological and texture features. They performed endogenous VIM-RFP labeling to observe the EMT process in lung cancer cells (Fig. 5b(i, ii)). Simultaneously, they recorded and quantified the cell body shape of VIM-RFP cells in the time-lapse images, using a deep learning-based image analysis algorithm (Fig. 5b(iii)).

Toneff *et al.* [68] generated a dual fluorescent reporter system by fusing green and red fluorescent proteins with miR-200 and E-cadherin, respectively (Fig. 5c). They used this system to robustly detect the EMT and MET processes in breast cancer cells through flow cytometry (Fig. 5c(ii)) and fluorescence microscopy (Fig. 5c(iii)). This dual fluorescent sensor potentially allows for the identification of minor subpopulations of cells with mesenchymal properties within epithelial-like cell populations and may more effectively distinguish cancer stem cell–like cells to a single sensor.

Overall, immunostaining and imaging enable the direct visualization and monitoring of the EMT process. However, most of the studies focused on cell line models, primarily targeting single markers, which makes it challenging to capture the dynamic molecular profiles of tumor cells. Further studies could expand to include multiple marker analyses to complete a comprehensive EMT trajectory analysis and visualize real-time single-cell changes during the EMT process. Despite the promise of these technologies, their clinical applications still require further development.

### Spatial transcriptomic

Spatial transcriptomics is a novel approach for investigating and visualizing gene expression patterns within the spatial context of tissue samples [69]. This technique provides a comprehensive understanding of gene activities across different regions of a tissue, enabling the visualization of interactions between different cell types, and the microenvironment within tissues [69]. Recent studies have utilized single-cell RNA sequencing (sc-RNA seq) coupled with spatial transcriptomic analysis to investigate the EMT score within various tumor tissues.

**Figure 6.**
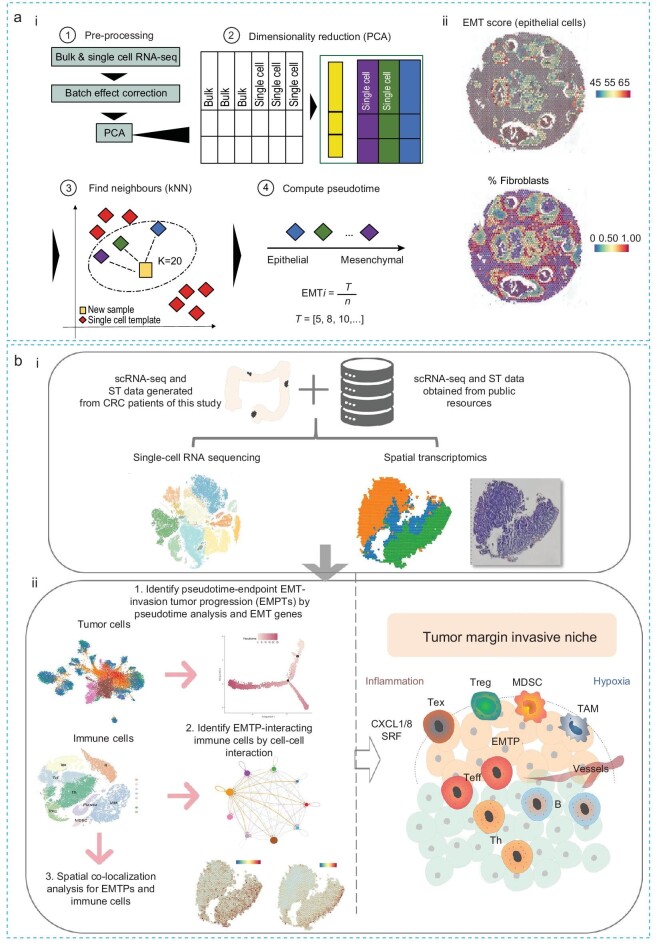
Spatial transcriptomic analysis. (a) (i) Constructing the EMT trajectories based on bulk/sc-RNA sequence dataset: ① process bulk and single-cell datasets; ② perform dimensionality reduction; ③ use the k-nearest neighbor (kNN) algorithm to map new samples onto the previous reference EMT trajectories; ④ sort tumors by mesenchymal potential using an EMT pseduotime axis. *T* = EMT value at the specific time point, *n* = number of neighbors for sample *i*. (ii) EMT scores and the fraction of fibroblasts are visualized within individual spots profiled across the tissue in a selected breast cancer slide, derived from spatial transcriptomics data from Patient 1. The color range indicates the expression of markers of the specific cell state (for EMT) and the fraction of cell types (for fibroblasts) [70]. Copyright 2023, Springer Nature. (b) (i) Combination of scRNA-seq and spatially resolved transcriptomics dataset to evaluate (ii) the EMT pseudo time trajectory and to investigate the spatial interactive relationship between EMT-invasive malignant tumors and immune cells in primary colorectal cancer tissues at different stages [71]. Copyright 2023, Springer Nature.

Tagliazucchi *et al*. [70] have employed a method to evaluate EMT transformation in individual tumors based on transcriptomic signals, aiming to reveal the molecular hallmarks associated with EMT intermediate states (Fig. 6a(i)). They analyzed 7180 tumors to reveal three macro-states along the EMT trajectory: epithelial, hybrid E/M, and mesenchymal phenotypes, discovering the prognostic and therapeutic implications across these states. They found that the hybrid E/M state is prevalent and heterogeneous, potentially serving as an intermediate stage in cancer progression. Furthermore, they leveraged spatial transcriptomic coupled with single-cell datasets to observe the interaction among tumor cells, immune cells, and fibroblasts within the tumor microenvironment (Fig. 6a(ii)). Overall, this study has comprehensively analyzed the multifaceted nature of the EMT trajectory in cancer and highlighted the intrinsic and microenvironmental factors in tumor progression and therapeutic response.

Wang *et al.* [71] also employed scRNA-seq coupled with spatial transcriptomics (Fig. 6b(i)) to investigate the EMT trajectory and the relationship between EMT-invasive malignant tumors and immune cells in primary colorectal cancer (CRC) tissues at different disease stages (Fig. 6b(ii)). They discovered remarkable spatial reprogramming of regulatory and immunosuppressive cells during the invasive and expansive process of the tumor. Specifically, in stage-I cancer tissues, a high inflammatory margin was observed at the invasive niche, while in stage-III tissue, a more hypoxic pre-invasive niche was produced. This study used advanced sequencing techniques to elucidate the spatiotemporal relationship between EMT-invasive malignant tumors and immune cells in CRC tissues at different stages. It highlights the impact of the tumor microenvironment, identifies prognostic genes, and improves the understanding of regulatory and immunosuppressive cells in cancer progression. Overall, these two studies employ advanced spatial transcriptomic analysis to unravel the relationship between tumor and immune cells, providing deep insights into the tumor microenvironment during the EMT process.

## EMERGING NANOTECHNOLOGIES FOR LIQUID BIOPSY BASED EMT MONITORING

Liquid biopsy has emerged as a powerful diagnostic approach for tracking EMT-related signatures in clinical settings. It is less invasive than tissue biopsy and allows for frequent and routine sample collection, making it ideal for real-time monitoring of EMT. This advantage is crucial for dynamically monitoring EMT and early detection of cancer metastasis. However, due to the low abundance of tumor-related biomarkers such as CTCs in circulation, it is essential to employ sensitive detection methods for accurate clinical monitoring.

Compared to conventional techniques that require large sample volumes and are invasive, emerging nanotechnologies provide several advantages, including higher sensitivity for detecting low-concentration biomarkers and minimal invasiveness. Additionally, some nanotechnologies allow for multiplexing detection of multiple targets simultaneously. These features allow nanotechnologies to offer improved precision and practicality for early EMT detection and monitoring [72,73]. The subsequent section discusses the latest developments in emerging nanotechnologies for investigating EMT and cancer metastasis. The reviewed emerging nanotechnologies are grouped into strategies for electrochemical biosensors, magnetic biosensors, surface-enhanced Raman spectroscopy (SERS), and other detection methods.

### Electrochemical biosensors

Electrochemical sensors utilizing nanomaterials as detection probes have demonstrated enhanced sensitivity, specificity, and rapid readout. These biosensors typically consist of three major components: a biorecognition element, a signal transducer, and an electrode [74]. The electrode can be functionalized with biorecognition elements, such as antibodies, enzymes, and synthetic molecules, to specifically capture target markers [75]. The presence of these target markers induce changes in electrical signals, which are subsequently detected by the signal transducer [76]. Since reactions are generally detected only in close proximity to the electrode surface, the performance of electrochemical biosensors is influenced by the electrode properties, including materials used, surface modification, and structure design [77].

**Figure 7.**
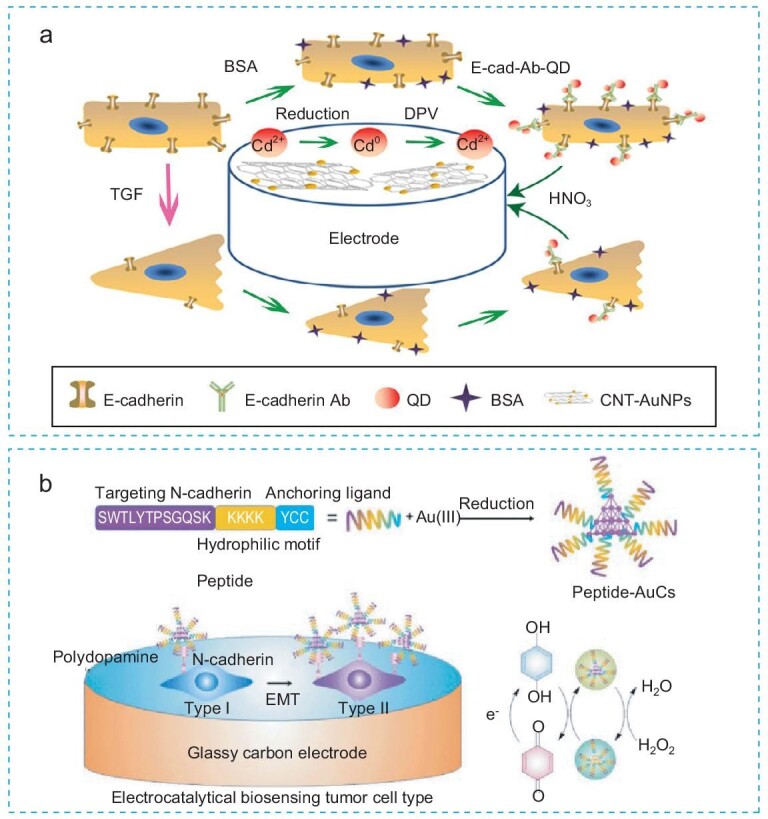
Electrochemical biosensors. (a) A dual readout electrochemical-fluorescence biosensor detects E-cadherin on lung cancer cells during the EMT process. Reproduced with permission from Ref. [37]. Copyright 2020, Springer Nature. (b) A metal cluster-based electrochemical sensing system for detecting N-cadherin on cancer cells during the EMT process. Reproduced with permission from Ref. [78]. Copyright 2021, American Chemical Society.

Du *et al.* [37] developed a dual readout electrochemical biosensor to monitor E-cadherin expression in lung cancer cell lines undergoing TGF-β-induced EMT. They utilized a carbon nanotube–gold nanoparticle (CNT-AuNP) modified electrode as the electrochemical sensing platform and CdSe/ZnS quantum dots (QDs) as fluorescence probes (Fig. 7a). Incorporating QDs into the electrochemical sensing system allowed simultaneous fluorescence detection alongside electrochemical signals, enhancing sensitivity and specificity in detecting changes of E-cadherin expression during the EMT process. In addition, the use of electrochemical detection methods eliminated the need for cell lysis, fixation, and secondary antibody incubation, saving time and reducing costs. However, future developments of this technology may face challenges due to the heterogeneity and plasticity of tumors during the EMT process, especially if they only detect a single marker.

Han *et al.* [78] engineered a metal cluster-based electrochemical biosensing system to detect EMT progression by quantitatively analyzing N-cadherin in pancreatic cancer cells (Fig. 7b). This system used a glassy carbon electrode (GCE) as a sensing platform and employed a peptide-modified gold cluster (AuC) as the detection probe. The AuC probe was synthesized through the reduction of peptide-anchored Au (III) to Au (0), followed by the formation of metal clusters. The peptide was designed with one end targeting N-cadherin specifically, while the other end anchored to Au (III) during synthesis. This innovative platform enables the sensitive detection of as few as 5000 tumor cells expressing N-cadherin, highlighting advancements in probe design and analytical methods for precise biomarker quantification in cancer research.

While both studies successfully developed proof-of-concept biosensors, they utilized cell line models for technology validation and did not include clinical samples. The sensitivity of their biosensors might be challenging to accurately measure the trace-level biomarkers in complex body fluids. Moreover, single-marker detection also limits technological applications in heterogeneous cancers and dynamic EMT processes.

### Magnetic biosensors

Magnetic biosensors have emerged as a novel platform for the detection, isolation, and identification of EMT biomarkers. These biosensors use a magnetic field to enrich biomolecules labeled with magnetic probes [79]. Advantages of magnetic biosensors include the stability of magnetic probes compared to optical probes like fluorescent tags, the absence of background noise effects, high sensitivity, and the ability to remotely measure and regulate the biosensor using an external magnetic field [79].

**Figure 8.**
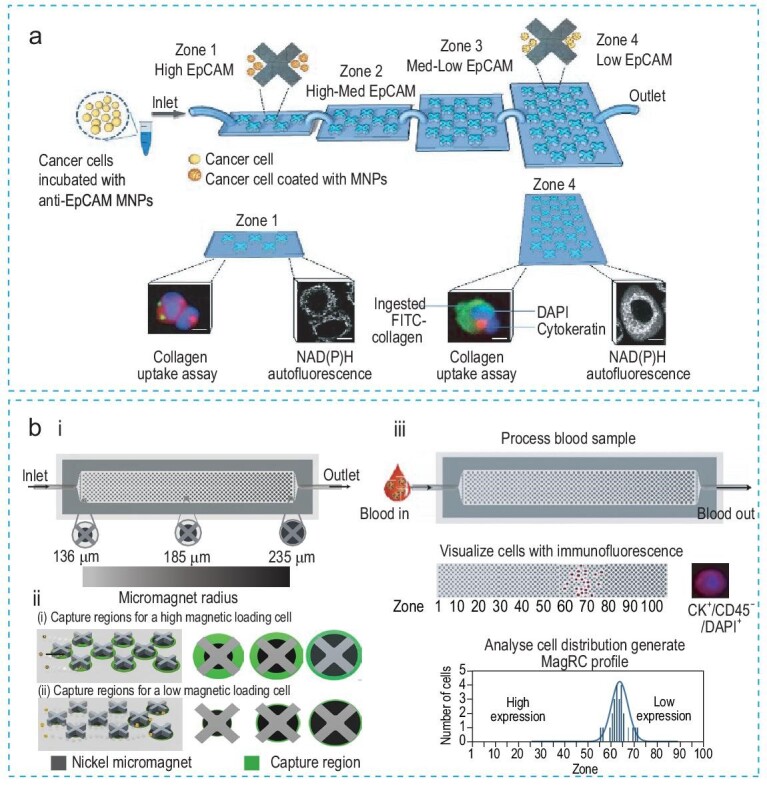
Magnetic biosensors. (a) The magnetic sorting device separates tumor cells based on their epithelial phenotypes. To evaluate the invasiveness of cells during EMT, the captured cells are released to analyze their collagen uptake and metabolic activities. Reproduced with permission from Ref. [80]. Copyright 2017, American Chemical Society. (b) (i) The MagRC microfluidic chip with 100 distinct zones containing varied magnetic capture zones. (ii) The capture zones for high and low magnetic loading cells. (iii) The MagRC system for profiling cells in blood. Reproduced with permission from Ref. [81]. Copyright 2016, Springer Nature.

Green *et al.* [80] developed a magnetic sorting device to isolate a specific subpopulation of tumor cells for investigating their invasiveness during the EMT process (Fig. 8a). In their study, cells were labeled with anti-EpCAM magnetic nanoparticles and subsequently sorted based on their EpCAM levels. The isolated cells were then released from the device and their collagen uptake and metabolic activity were analyzed. However, it is important to note that the exclusive reliance on the EpCAM marker could potentially obscure the detection of other crucial phenotypic modifications integral to the EMT and the progression of tumorigenesis.

Similarly, Poudineh *et al.* [81] developed a magnetic ranking cytometry (MagRC) to track the EMT by CTC phenotyping (Fig. 8b(i–iii)). MagRC contains micro-engineered structures, including an x-shaped microstructure and nickel micromagnets. These structures provide slow flow for capturing cells with magnetic nanoparticles and enable discretized sorting of subpopulations. In their study, MagRC sorted CTCs into 100 distinct magnetic zones based on their magnetic loading against surface proteins (Fig. 8b(i, ii)). Most importantly, their technology was applied to whole blood samples (1 mL) and was capable of isolating and profiling as few as 10 CTCs using EpCAM as the captured marker, outperforming the commercial CellSearch assay. They were able to track the EMT process using MagRC based on the downregulation of EpCAM on CTCs. To transfer this technology to clinical applications, they evaluated MagRC on prostate cancer patient samples for capturing CTCs. They noted a reduction in epithelial phenotypes in patients at advanced stages of the disease, indicating the occurrence of the EMT process.

In summary, magnetic biosensors show high potential for capturing CTCs and monitoring cancer metastasis. However, one of the major limitations of magnetic biosensors is their reliance on single-marker capture, particularly EpCAM in CTC detection, which may introduce a selection bias and result in the loss of important clinical information.

### SERS-based biosensor

SERS-based biosensors are a powerful analytical technique for analyzing liquid biopsies, including the detection of EMT-associated biomarkers. SERS signals are generated from molecules situated on or near the surface of plasmonic nanomaterials. When these nanomaterials are irradiated with a laser, they induce localized surface plasmon resonance, which enhances the electromagnetic field and amplifies the Raman signals [82]. SERS-based biosensors offer high sensitivity, multiplexing ability, and selectivity, making them highly effective for detailed molecular analysis of EMT biomarkers in liquid biopsy applications [83].

**Figure 9.**
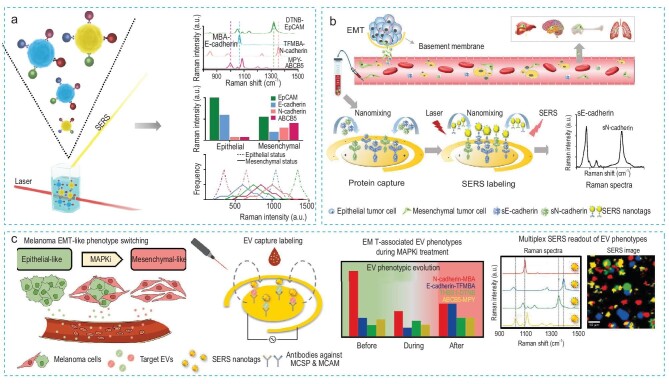
SERS-based biosensor. (a) A multiplex SERS barcoding system for profiling EMT-related phenotypic evolution on CTCs. Reproduced with permission from Ref. [84]. Copyright 2021, American Chemical Society. (b) A microfluidic-immunoSERS platform for EMT detection through monitoring the ratio shift of soluble N-/E-cadherin proteins. Reproduced with permission from Ref. [85]. Copyright 2020, John Wiley and Sons. (c) A microfluidic-immunoSERS platform for tracking EMT-like EV phenotypic switching during MAPKi therapy. Reproduced with permission from Ref. [87]. Copyright 2024, Elsevier.

Zhang *et al.* [84] developed a multiplex SERS barcoding system to profile EMT-associated phenotypes on CTCs from patients’ blood (Fig. 9a). They synthesized SERS barcodes by conjugating antibodies to gold nanoparticles patterned with Raman reporters, which generated characteristic Raman peaks. Using this multiplex SERS barcoding system, they could simultaneously profile two epithelial markers (E-cadherin and EpCAM) and two mesenchymal markers (N-cadherin and ATP-binding cassette sub-family B member 5 (ABCB5)) on CTCs. They observed the downregulation of epithelial markers and upregulation of mesenchymal markers with heterogeneous evolution on CTCs in late-stage breast cancer patients.

To non-invasively track the EMT process, Zhang *et al.* [85] also engineered a microfluidic-immunoSERS platform to detect soluble N-/E-cadherin proteins shed from tumor cells into serum (Fig. 9b). These soluble N-/E-cadherin proteins were captured by antibodies functionalized on gold electrode surfaces and detected by the duplex SERS barcoding system. To enhance the sensitivity and specificity of assays, they applied alternating current electrohydrodynamic (ac-EHD) nanomixing on the gold electrode surfaces. Integrating the ac-EHD nanomixing and SERS nanotags, they successfully detected soluble N-/E-cadherin proteins from as few as 10 cells/mL in simulated patient samples. They observed high soluble N-cadherin and low E-cadherin in serum from late-stage breast cancer patients compared to healthy individuals, suggesting the potential capability of monitoring the EMT process in blood.

EVs carry proteins and nucleic acids, reflecting the cell of origin. Compared to CTCs, EVs have higher concentrations in blood, making EVs a promising biomarker for tracking the EMT process [86]. Zhou *et al.* [87] adapted the microfluidic-immunoSERS platform for phenotypic characterization of EMT-associated phenotypes on circulating EVs (Fig. 9c). They longitudinally monitored 8 melanoma patients receiving mitogen-activated protein kinase inhibitor (MAPKi) treatment and observed EMT-like phenotypic evolution on EVs during treatment.

In conclusion, SERS-based biosensors integrated with liquid biopsy biomarkers hold great potential for non-invasively monitoring EMT. However, their clinical translation is currently hampered by limitations such as unsatisfactory quantification ability and the requirement for specialized laboratory skills.

### Other nanotechnologies

**Figure 10.**
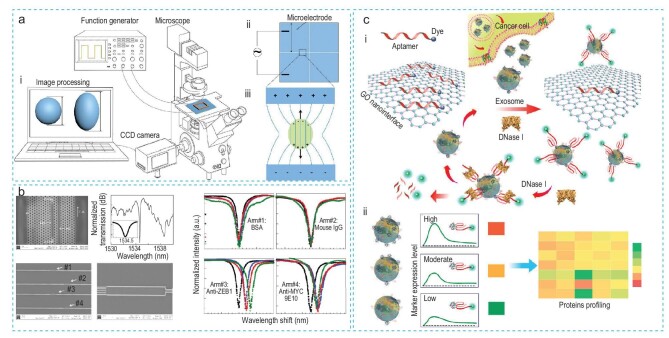
Other nanotechnologies applied to detect EMT. (a) Quantifying the mechanical deformability of cancer cells during the EMT process. (i) Schematic for running the microchip to detect EMT. (ii) The illustration of the microelectrode. (iii) The measurement of elasticity and viscosity of cells. Reproduced with permission from Ref. [88]. Copyright 2017, Elsevier. (b) The label-free photonic crystal microcavity biosensor in silicon-on-insulator detects the EMT transcription factor ZEB1. Reproduced with permission from Ref. [89]. Copyright 2013, Elsevier. (c) The aptamer nanoprobe-based profiling platform. (i) The GO nanointerface recognizes aptamer-targeted exosomes secreted from cancer cells. This is followed by incubation with DNAase I to expose exosome protein markers for another round of aptamer-binding, thereby inducing signal amplification. (ii) Exosome protein profiling. Reproduced with permission from Ref. [90]. Copyright 2018, American Chemical Society.

Other nanotechnologies have also been developed to investigate changes in the deformability of CTCs during EMT, EMT transcription factors, and EV surface proteins. Teng *et al.* [88] established a microchip for quantifying the mechanical deformability of cancer cells during EMT using an electrode-based approach (Fig. 10a). They designed the microchip with electric wires connected to indium tin oxide electrodes with a 20-µm gap, generating a non-uniform electric field for stretching single cells with di-electrophoretic forces. Their measurements distinguished between epithelial and mesenchymal cells, showing that mesenchymal cells were less elastic and viscous than epithelial cells after TGF-β treatment. Similarly, Chakravarty *et al*. [89] developed a photonic crystal (PC) microcavity biosensor to detect the EMT transcription factor ZEB1 in NCI-H358 cell lysates (Fig. 10b). In this setup, a shift in resonance wavelength indicated the binding of corresponding antigens to the PC microcavity. Both of these methods utilize label-free technology to analyze tumor cell properties during the EMT process and have the potential to translate into clinical applications.

Furthermore, Jin *et al.* [90] established an aptamer nanoprobe-based profiling platform to phenotypically monitor exosome surface protein levels during the EMT process in prostate cancer (Fig. 10c). The platform integrated graphene oxide (GO) with target-responsive aptamers and enzyme-assisted exosome recycling, to profile exosomes derived from five cell types, revealing a heterogeneous exosome phenotypic pattern. The assay demonstrated a detection limit of 1.6 × 10^5^ exosomes/mL. Following TGF-β treatment of prostate cancer LNCaP cells, they observed that the ratios of EpCAM to prostate-specific membrane antigen (PSMA) changed from 4-fold to 0.5-fold, suggesting the initiation of EMT transition. Additionally, higher levels of EpCAM and PSMA were observed on exosomes from 14 prostate cancer patients compared to healthy individuals, showing potential for cancer diagnosis and treatment monitoring.

## OUTLOOK AND CONCLUSION

Monitoring the EMT process is crucial for understanding the onset of cancer metastasis. Numerous studies have explored its mechanisms and discovered new EMT-related markers using both conventional and advanced biomolecular approaches, such as single-cell RNA sequencing, mass spectrometry, and spatial transcriptomic analysis. Despite these advancements, dynamic monitoring of the EMT process through liquid biopsy is still in its early stages. This is primarily due to the low abundance of EMT-associated markers in complex bodily fluids, necessitating the development of highly sensitive and specific technologies.

Emerging nanotechnologies, including electrochemical, magnetic, and SERS-based nanotechnologies, offer significant promise due to their high sensitivity, specificity, and multiplexing capabilities. However, several challenges must be overcome before they can be routinely used in clinical practice:

Validation with real patient samples: Most studies use simulated patient samples to evaluate nanotechnology performance, which may not fully represent clinical conditions. To overcome this, future developments should focus on validating these nanotechnologies in large clinical cohorts to ensure their effectiveness with actual patient samples and meet clinical requirements.

Automation and user-friendly platforms: Current nanotechnologies often require specialized laboratory skills, limiting their accessibility in clinical settings. Future developments will aim to improve automation and create user-friendly platforms to make these nanotechnologies more accessible and easier to use in routine clinical practice.

On-site analysis capabilities: Existing nanotechnologies are frequently not designed for rapid, on-site analysis, which is crucial for timely decision-making. Miniaturization and integration of these platforms with point-of-care devices will enable rapid, on-site analysis, providing real-time data for more informed treatment decisions.

Data analysis: The complex data generated by nanotechnologies can be challenging to interpret using traditional methods. The application of artificial intelligence to analyze complex data represents another exciting outlook. These technologies can uncover patterns and correlations that might not be apparent through traditional analyses, assisting in identifying markers and predicting EMT progression.

In conclusion, successfully transitioning EMT studies to clinical applications will require addressing current limitations through technological innovation and streamlined workflows. Combining both traditional approaches for molecular discovery with advanced technologies for real-time monitoring of the dynamic EMT process will be essential. As these technologies evolve, they are expected to become indispensable tools in the early detection, monitoring, and management of cancer, ultimately improving patient outcomes and advancing personalized medicine.
